# Exploring the genetic factors behind the discrepancy in resistance to bovine tuberculosis between African zebu cattle and European taurine cattle

**DOI:** 10.1038/s41598-024-52606-2

**Published:** 2024-01-29

**Authors:** SangJung Lee, Charton Clémentine, Heebal Kim

**Affiliations:** Department of Agricultural Biotechnology and Research Institute of Agriculture and Life Sciences, Seoul National University, Seoul, 08826 Republic of Korea

**Keywords:** Agricultural genetics, Population genetics

## Abstract

Caused by the pathogenic agent *Mycobacterium bovis*, bovine tuberculosis (bTB) is a major concern in cattle breeding due to both its zoonotic potential and economic impact. Greater resistance to this disease has been reported in certain African zebu breeds compared to European taurine breeds. However the genetic basis for the lower susceptibility to bTB infection observed in zebu cattle remains poorly explored. This study was conducted on whole genome sequencing data of three bTB infection-resistant African zebu breeds and two bTB infection-susceptible taurine breeds to decipher the genetic background. A set of four selection signature statistics based on linkage disequilibrium, site frequency spectrum, and population differentiation were used on SNPs whereas between population variance based VST and t-test were used on CNVs. As a complement, genes from previous literature reported as candidate genes for bTB resistance were also inspected to identify genetic variations. Interestingly, the resulting nine candidate genes had deleterious missense variants (*SHC3, IFNGR1, TLR2, TLR6, IL1A, LRRK2, EP300* and *IRAK4)* or a CNV difference (*CD48*) segregating between the groups. The genes found in the study play a role in immune pathways activated during *Mycobacterium* infection, contributing to the proliferation of immune cells and the granuloma formation, ultimately modulating the outcome of the infectious event. In particular, a deleterious variant in the *LRRK2* gene, whose deficiency has been linked to improved prognosis upon tuberculosis infection, was found in the bTB infection-resistant zebu breeds. Therefore, these genes constitute credible candidates in explaining the discrepancy in *Mycobacterium bovis* infection susceptibility among different breed.

## Introduction

With around two million deaths reported annually, tuberculosis (TB) remains to be the world’s deadliest infectious disease. In developing countries 10 to 15% of human TB cases are estimated to be caused by the bovine pathogenic agent *Mycobacterium bovis*^1^. To date, bTB remains a major public health threat and a source of substantial economic loss in cattle breeding.

*Mycobacterium bovis* is part of the wider *Mycobacterium tuberculosis* complex (MTBC). Though it was originally believed that cattle transmitted the causative agent to humans, today’s evidence show that it was more likely the other way around^2,3^. Transmission was thought to have occurred during domestication, around 10,000 years ago, but a recent study from 2020 provides a more modern (3rd to twelfth century AD) estimation for *Mycobacterium bovis* origin in East Africa^4^.

Though the prevalence of bTB has been shown to be determined by cattle management, evidence also point to individual genetic variation in cattle resistance to bTB infection^5^. Bovine tuberculosis susceptibility appears to be a trait of moderate heritability, ranging from 0.21 to 0.37^6,7^. Resistance to infection by disease-causing bacteria of the MTBC appears to be under polygenic control in numerous species^8^. Fewer TB infection resistance-related loci have been identified compared to other complex diseases when implementing GWAS^9^. Accordingly, bTB infection resistance studies, among which are ^10,11^, also revealed few candidate genes. Some of these genes could be related to non-specific immunity, particularly the ones involved in the defense of the respiratory tract (for example genes encoding proteins regulating bronchial mucus secretion or non-specific macrophages in lungs) and might participate in the elimination of a low dose bTB contamination^5^. However, the genes tend not to replicate between studies.

On the other hand, cattle sub-species also influence resistance to bTB infection^12,13^. Zebu (*Bos indicus*) breeds were historically shown to exhibit a higher resistance towards bTB than Taurine (*Bos taurus*) breeds^14–17^. Later reports show a higher prevalence of bTB in the European Taurine (EUT) Holstein breed than in African Zebu (AFZ) (mainly Arsi breed) or AFZ × EUT crosses (22.2,11.6 and 11.9% respectively) by conducting a comparative intradermal tuberculin test with injection of purified protein derivatives of *Mycobacterium bovis* on a total of 5,424 cattle^13^. In the same study, lesion severity scores of lungs and lymph nodes (according to the scoring procedure developed by Vordermeier et al.^18^) showed a significant difference among breeds. Subsequently, Vordermeier et al.^19^ found the AFZ Boran breed calves to be less susceptible to bTB infection than the EUT Holstein calves were. Moreover, after developing the disease, the severity of the lesions was significantly lower in indicine individuals than in Holstein. The indicine individuals were also found to derive more protection from the BCG Bacillus Calmette-Guerin (BCG) vaccination^16^. Also, previous studies show that bTB has a geographical origin overlapping with the long time habitats of the more resistant breeds, which coincides with our basis of the study^4,20^ (Fig. S1).

Because bTB appeared after the divergence between *Bos indicus* and *Bos taurus*, the higher resistance to bTB in zebu breeds may be due to one or more of the three following scenarios : (1) a recent (less than 2000 generations) and ongoing selection towards bTB infection resistance, (2) an ancient selection in immune genes due to exposure to various pathogens, occurring in one of the two sub-species and conferring by chance a pre-adaptation against bTB infection in zebu or (3) a random genetic drift alone that led to alleles more adapted towards resistance to bTB infection by chance. While *Bos indicus* breeds have been shown to exhibit a higher resistance to bTB infection than *Bos taurus* breeds, only one study aiming at identifying the genes related to this resistance was conducted in *Bos indicus* × *Bos taurus* crosses^11^. However, due to the use of low-density markers, only one loci associated to the Toll-Like Receptor (TLR) complex was discovered. The causal mutations at the origin of the bTB infection resistance discrepancy between *Bos indicus* and *Bos taurus* breeds thus remains vastly unknown.

This study aimed to decipher the genetic basis for the differences in bTB susceptibility to infection at high resolution using WGS data from three African zebu breeds reported as more resistant to bTB infection from the aforementioned studies (Arsi, Ethiopian Boran and Kenyan Boran) and two bTB infection susceptible breeds (Holstein, and Jersey). In order to gain maximum insight, this study proceeded in two complementary steps. The first step identifies genomic regions related to immunity having undergone selection. The second step explores genomic regions already reported in the literature as modulating resistance to bTB infection.

## Results

### Alignment statistics

The CNVs calling accuracy has been shown to highly depend on the coverage and sequencing depth. Previous studies reported that a 5 × depth coverage was sufficient for CNV detection^21–23^, with accuracies as high as 97% being reached for a depth > 8 ×^23^. The minimum and average mean depths reached in our study being 7.9 × and 9.4 ×, respectively, with associated alignment rate and coverage of 99.3 and 95.0% (Table. S2), we estimated that CNVs calling accuracy was sufficient.

### Population structure

Population structure of the five breeds (30 individuals in the Resistant (Res) group and 20 individuals in the non-resistant (NRes) group) was examined to decipher the genetic distance between and within each group.

**Figure 1:**
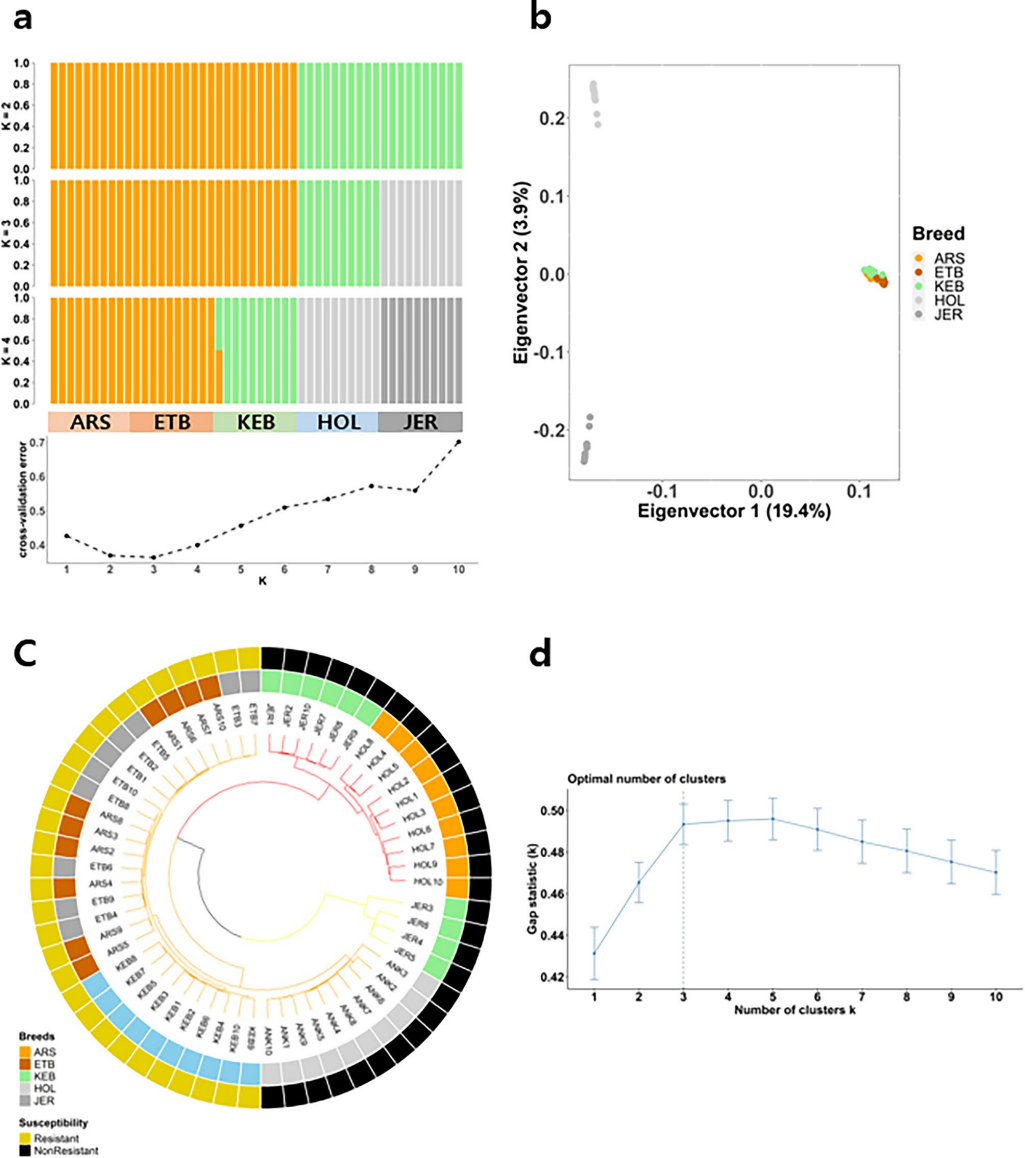
(**a**) Admixture analysis result for K 2–4 and cross-validation error plot. (**b**) PCA result for the 50 samples used in the study. (**c**) Dendrogram according to the copy number differences among individuals. (**d**) Gap statistic plot indicating the most probable K for clustering.

The number of ancestries was then estimated using the Admixture software^24^. As evidenced from the cross-validation error estimation plot (Fig. 1a), three ancestral populations seemed the most sensible modeling choice considering the current dataset. The first hypothesis (three ancestral populations) isolates Res groups and NRes groups from each other while the second hypothesis (two ancestral populations) isolates NRes (Holstein and Jersey) from Res breeds. The split identified between the NRes and Res breeds using the pairwise fixation index (FST > 0.19, Table 1) was slightly higher than the FST between two NRes breeds (0.16). On the other hand, a low (< 0.03) FST was observed between the three Res breeds, indicating a closer genetic composition within the Res group than within the NRes group.

**Table 1:** Pairwise Weir and Cockerman weighted FST computed between each of the five breeds: Arsi, Ethiopian Boran, Kenyan Boran, Holstein, and Jersey.

	Arsi	Ethiopian Boran	Kenyan Boran	Holstein
Ethiopian Boran	0.00303			
Kenyan Boran	0.02085	0.01896		
Holstein	0.19126	0.20201	0.20822	
Jersey	0.20327	0.21396	0.22126	0.16426

PCA eigenvalues evidenced a substantial jump between the first and the second dimensions. The first dimension of the PCA explained 19.4% of the variability in the dataset, while the second dimension explained 3.9% of the variability. As illustrated on (Fig. 1b), dimension 1 clearly separated the three African zebu breeds (Arsi, Ethiopian Boran, and Kenyan Boran) from the two European taurine breeds (Holstein and Jersey), whilst the second dimension segregated between the Holstein and Jersey breeds.

The CNV-based distance was determined by drawing a dendrogram based on the CNV difference between individuals (Fig. 1c). Similarly, to what was observed in the SNP-based population structure results, the Res breeds clustered together while some of the Jerseys in the NRes breeds seemed to be grouped separately. Identical to the SNP based methods, 3 clusters were recommended as the optimal population number from the results of the Gap statistics (Fig. 1d).

### Candidate genes issued from the selection signature analyses

The six complementary methods (4 SNP based methods and 2 CNV based methods) implemented in the present study correspond to different selection signatures and are associated with various time scales. By annotating the upper 1% window of the empirical distribution obtained for each method, a total of 783, 882, 371, 619, 37, and 303 genes were identified as under recent or ongoing selection using the FLK, FST, XP-EHH, XP-CLR, VST, and t-test methods, respectively.

As for the SNP based methods, 635 genes were found to replicate for at least two methods. Among these genes, a total of 165 genes were found using three or more methods and 18 genes (*ABLIM3, ADGRV1, AFAP1L1, BBS9, BBX, CLCC1, CMSS1, COL8A1, CSNK1A1, CTSZ, FILIP1L, GRPEL2, MED27, MSRB3, NELFCD, REEP1, TFEC,* and *ZNF831*) were found common to 4 out of the 4 SNP based methods. A total of 35 genes were found to replicate in the two CNV-based methods (Table S4).

The DAVID gene ontology analysis was implemented on every gene from the six methods above and resulted in the finding of 268 significant GO terms (GO:BP 114, GO:CC 71, and GO:MF 83) and 60 KEGG pathways. Resistance to bTB solicits both innate and adaptive immunity responses. The innate immunity of lung alveolar epithelial cells in response to *Mycobacterium* exposure plays a crucial role in eliminating the pathogen. T-cells, in turn, act as a regulatory force, mitigating the overabundance of pro-inflammatory factors that could otherwise lead to immunopathology. As a result, we chose to thoroughly explore all GO terms and KEGG pathways associated with relevant immune responses, through a comprehensive literature review. The most pertinent terms were “Bacterial invasion of epithelial cells”, “T cell receptor signaling pathway”, “Leukocyte trans endothelial migration”, “Negative regulation of T cell proliferation”, “Negative regulation of leukocyte cell–cell adhesion”, “Apoptosis”, “Cell death”, and “Endocytosis”. Many other terms, involved in protein adhesion, interaction and transportation, that could be potentially related to resistance to *Mycobacterium*, were also found. In total 282 genes were investigated, among which are 242 SNP-based genes and 40 CNV-based genes (Table S3).

**Figure 2:**
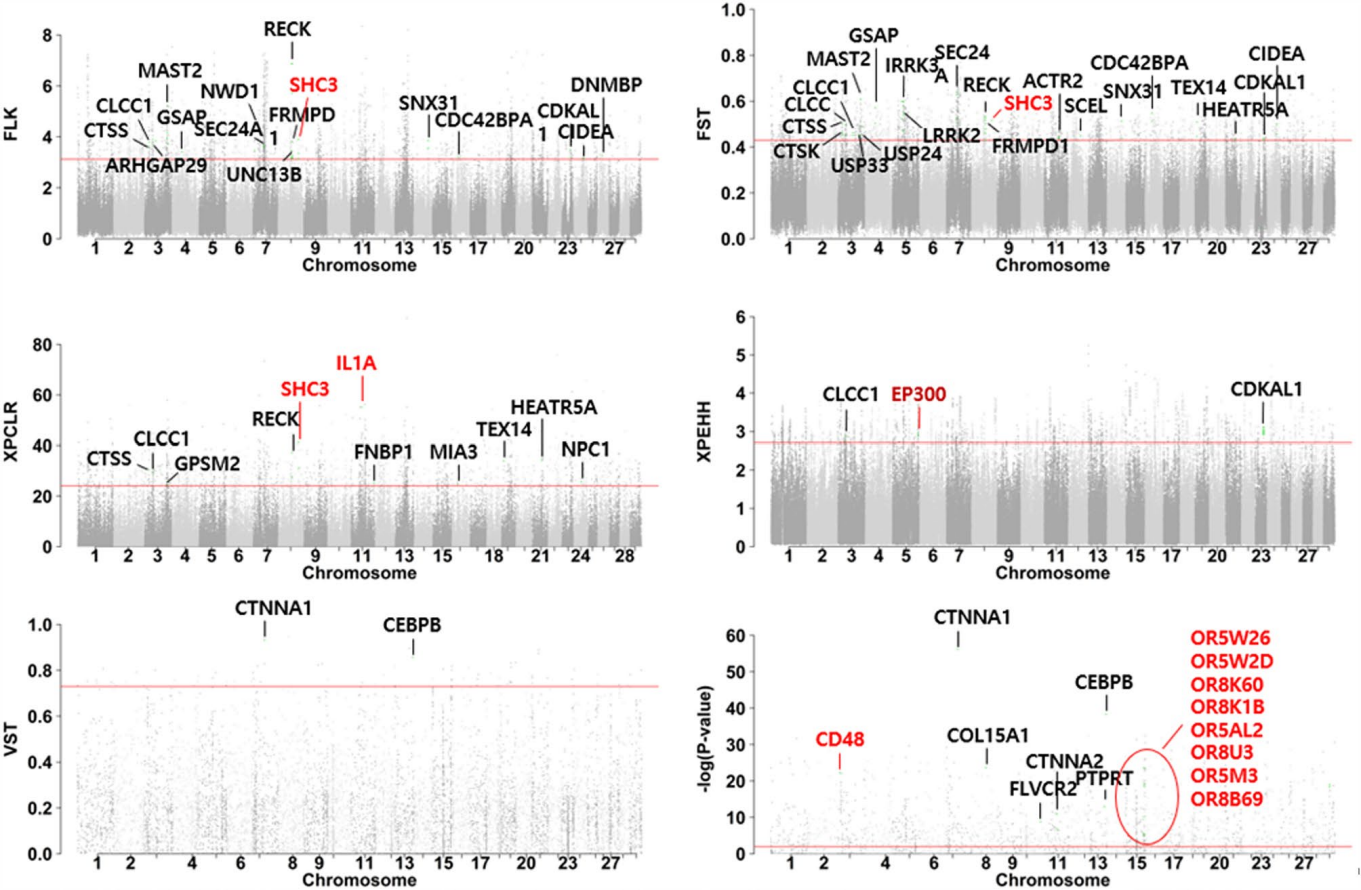
The score values for each window selected from the selection signature analyses (FLK, FST, XP-CLR, XP-EHH, VST, t-test) used in the study. The horizontal line in red shows the 0.01 significance level of the scores without binning. Labeled dots are the highest scoring windows in each gene. Genes labeled in red are the strongest candidate genes chosen from the study.

**Figure 3:**
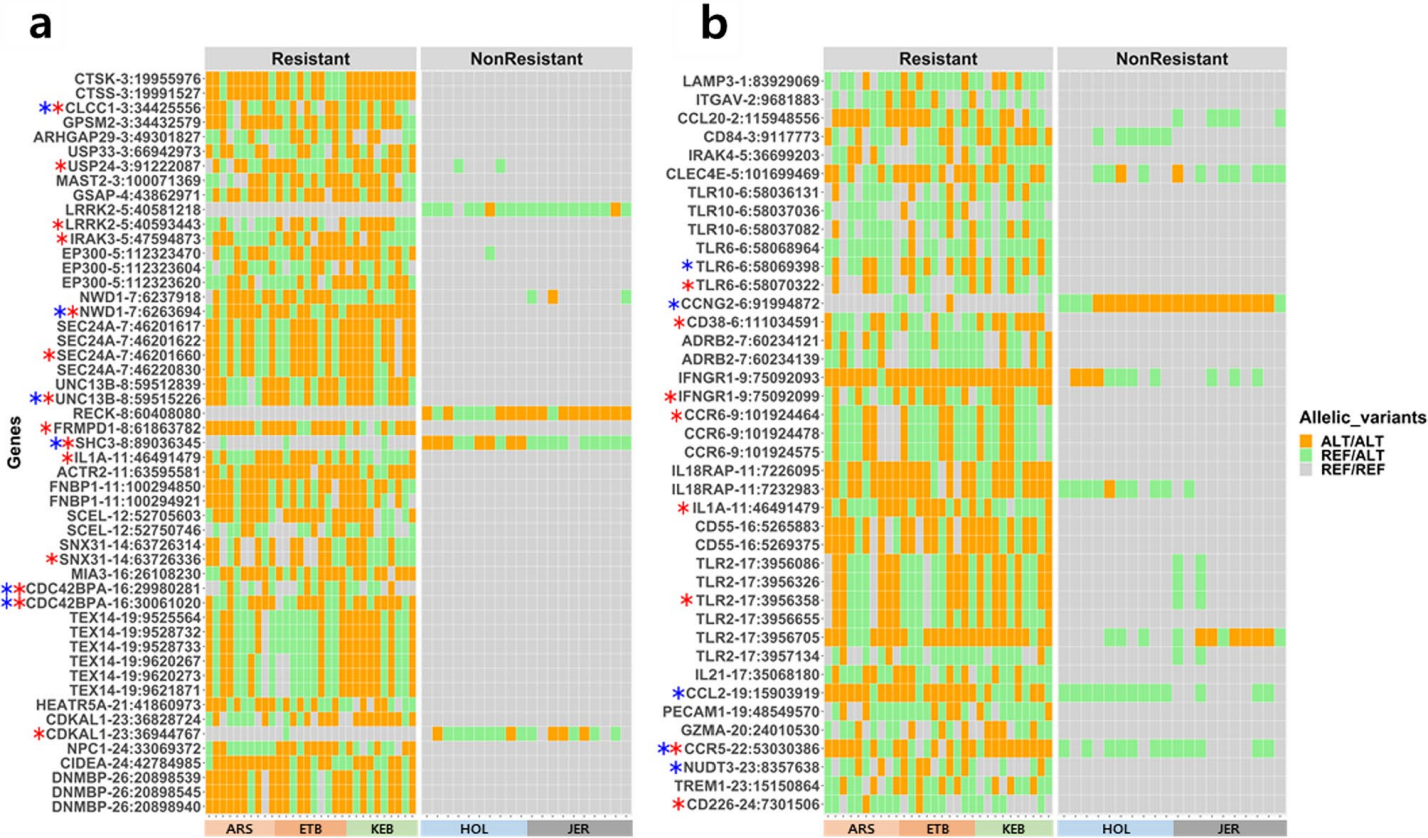
(**a**) Missense variants filtered after the selection signature analysis and gene ontology analysis. (**b**) Missense variants filtered after the literature review and gene ontology analysis. Blue and red asterisks indicate the significance in SIFT score (< 0.05) and PROVEAN score (< − 1.3) respectively.

The 242 SNP-based genes were inspected for the presence of missense variants segregating between the Res and NRes populations. The resulting 32 genes are displayed in (Figs. 2 and 3a). These missense variants were further filtered based on whether they were estimated as having a deleterious effect on the protein formation (Table 2). The *CDC42BPA, CLCC1, FNBP1, NWD1, SCEL, SHC3, TEX14*, and *UNC13B* missense variants were estimated as deleterious by the SIFT score and the *CDC42BPA, CDKAL, CLCC1, FRMPD1, IRAK3, LRRK2, NWD1, SEC24A, SHC3, SNX31, UNC13B* and *USP24* variants were found deleterious by the PROVEAN score.

**Table 2:** Missense variant allele frequencies of the most serious candidate genes. SIFT score < 0.05 and Provean score < − 1.3 were considered as significant.

Gene, position (ref/alt)	AFZ alt	EUT alt	SIFT	Provean
*SHC3*, chr8:89,036,345 (C/T)	0.07	0.675	0	− 4.197
*IFNGR1*, chr9:75,092,099 (C/A)	0.67	0	0.13	− 2.2
*TLR2*, chr17:3,956,358 (A/T)	0.683	0.05	0.23	− 1.301
*TLR6*, chr6:58,069,398 (T/C)	0.65	0	0.04	− 0.399
*TLR6*, chr6:58,070,322 (T/C)	0.47	0	0.06	− 5.737
*IL1A*, chr11:46,491,479 (C/G)	0.717	0	0.2	− 2.159
*LRRK2*, chr5:40,593,443 (C/T)	0.6	0	0.07	− 3.803
*EP300*, chr5:112,323,620 (G/A)	0.7	0	0.26	− 0.248
*IRAK4*, chr5:36,699,203 (G/A)	0.483	0	0.25	− 0.559

**Figure 4:**
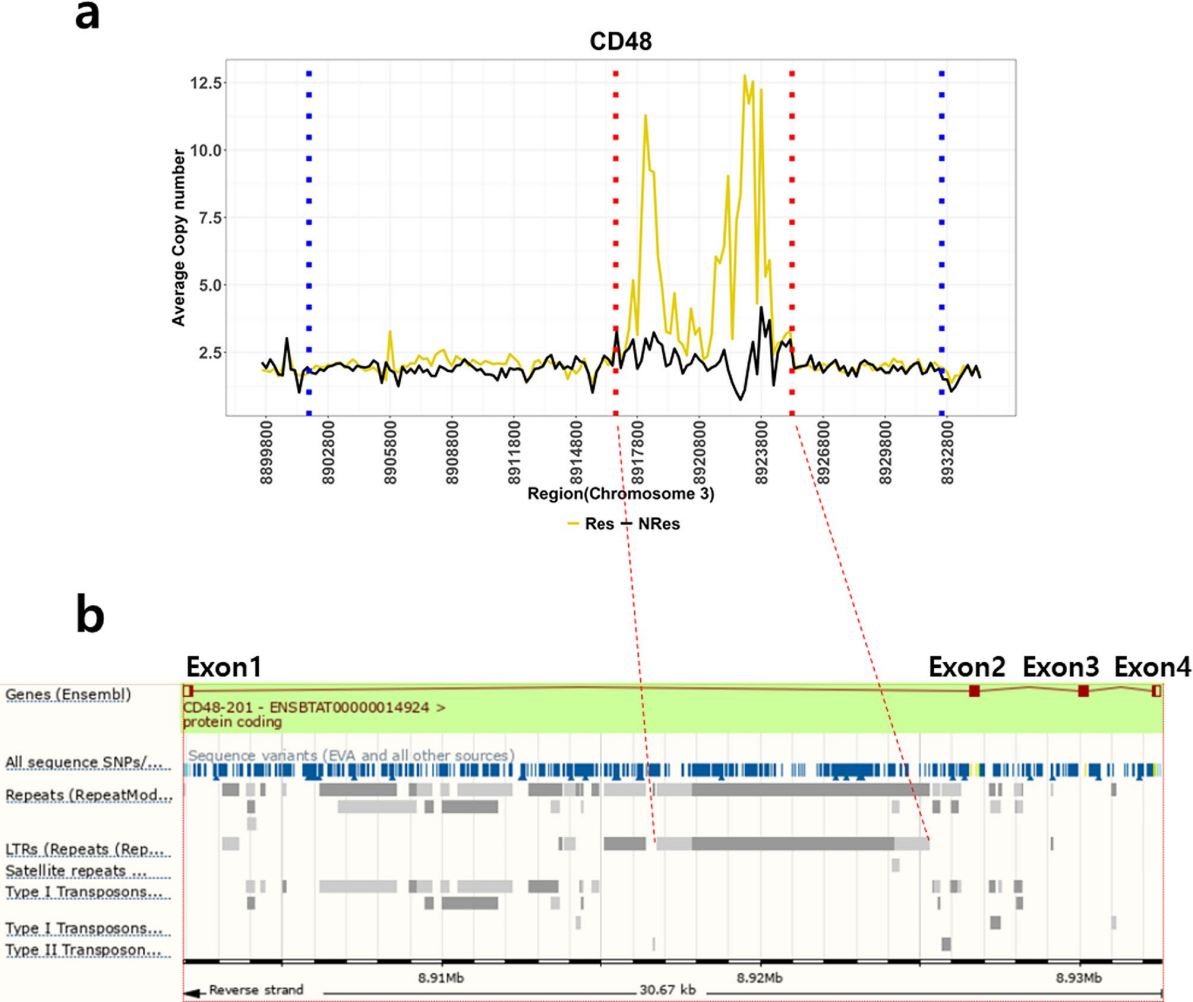
(**a**) Average copy number differences of every 200 bp regions were visualized as a line graph between Res and NRes in *CD48* (chromosome 3:8,901,877–8,932,543) with flanking regions of 2 kb on both sides. The x and y axes indicate the genomic region and average copy number, respectively. (**b**) Region of repeat was shown by Ensembl Genome browser. Repeats were called with Repeat Masker and Repeat Modeler. Region in-between red dotted lines indicate the overlapping repeat region which is mostly LTR (long tandem repeats). Blue line is the starting position of flanking regions.

The 40 CNV-based genes were further inspected for copy number differences within the gene. Remarkably, the *CD48* gene exhibited a copy number difference of up to 10 in certain intronic regions (Fig. 4a and b).

### Candidate genes issued from the literature

A total of 239 genes were considered when implementing the second strategy, i.e. exploring the genomic difference in the genes already reported in the literature as having potential relation to resistance to bTB infection. Among the 239 genes investigated, 25 genes exhibited missense variants that contrasted to some extent between the groups. The *CCL2, CCNG2, CCR5, NUDT3*, and *TLR6* genes were identified as deleterious by the SIFT score and *CCR5, CCR6, CD226, CD38, IFNGR1, IL1A, TLR2*, and *TLR6* genes were chosen by PROVEAN score (Fig. 3b).

In the final step, we restricted the most serious candidate genes to the ones presenting a clear causative relationship to bTB resistance according to the literature: *SHC3, IFNGR1, TLR2, TLR6, IL1A, LRRK2*, *CD48, EP300* and *IRAK4.* The *SHC3* missense variant stood out as the sole gene variant found in the NRes breeds, whereas missense variants were exclusively identified in the Res breeds for *IFNGR1*, *TLR2*, *TLR6*, *IL1A*, *LRRK2*, *CD48*, *EP300*, and *IRAK4* genes (Table 2).

## Discussion

*Mycobacterium* has successfully developed the ability to sustain latent and persistent infections by limiting the development of adaptive immune responses^25,26^. Accordingly, innate immunity is the key to determining the disease outcome upon *Mycobacterium* exposure. Several components of the innate immune system contribute to the pathogenesis of *Mycobacterium* infection, especially macrophages but also dendritic cells, neutrophils, mast cells, and NK (natural killer) cells. The recognition and phagocytosis of the bacteria are essential for the initiation of pro-inflammatory responses. Recognition is mediated through different receptors (toll-like receptors, nuclear oligomerization domain receptors, C-type lectin receptors and mannose receptor families^27,28^) while the inflammatory response appears to be mainly mediated through TLRs and possibly dectin-1^29–31^. Inflammation is a key process that can locally resolve TB infection via the recruitment of macrophages, neutrophils, NK cells, and eventually T-cells^32^ . In turn, these cells clear invading pathogens or can control bacilli growth while containing them within granulomas^33^. It is thus probable that tuberculosis sensitivity is modulated by both the host *Mycobacterium* recognition and the balance between pro- and anti-inflammatory factors. Indeed, weak inflammatory responses are reported to lead to mycobacterial infection propagation while strong inflammatory responses are likely to induce immunopathology^34^. Part of this management is thought to be genetically determined as it was reported in diverse species^34–37^.

**Figure 5:**
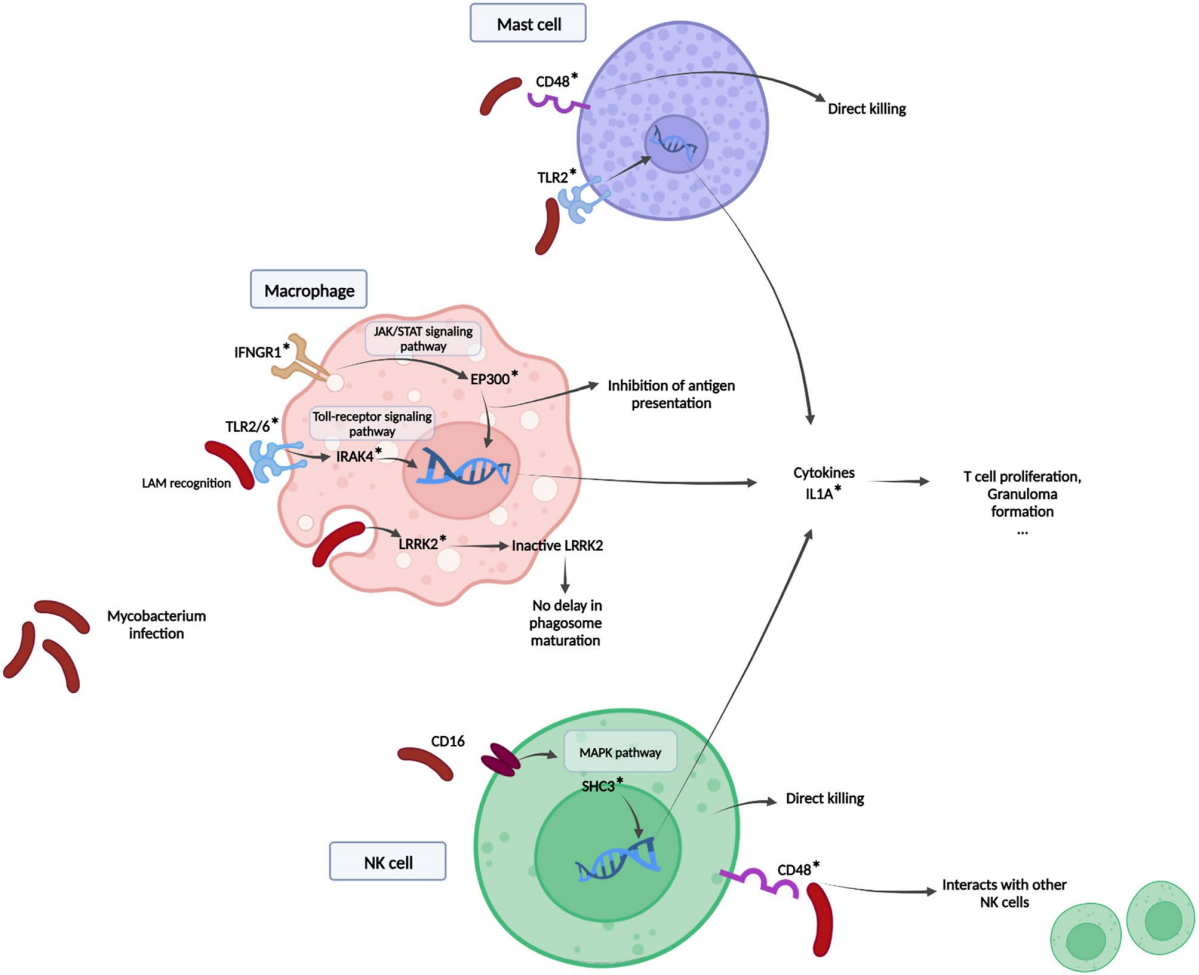
Involvement of the candidate genes in innate immune signaling after *Mycobacteria* encounters the immune cells. Genes presenting at least one missense variant in one of the two breed groups are identified with an asterisk, red in case this variant is probably deleterious (based on SIFT and Provean) score. The figure was drawn with Photoshop CC 2022 (https://www.adobe.com/products/photoshop.html).

In this study, we found evidence of differentially selected genes between the Res and the NRes breeds in several of the genes related to the pathways of known tuberculosis responses and NK cell-mediated cytotoxicity (Fig. 5). Among these genes, the most serious candidates were *SHC3, IFNGR1, TLR2, TLR6, IL1A, LRRK2* and *CD48*. These genes harbored deleterious missense variants which allelic frequencies contrasted between Res and NRes individuals. In addition, we also considered the *EP300* and *IRAK4* genes, which scores were close to being significantly deleterious, as putative candidate genes. We describe the relationship between these genes and the immune pathways modulating bTB resistance.

A strong candidate gene for the discrepancy in bTB infection resistance found in this study is the *LRRK2* gene (Leucine-rich repeat kinase 2). Indeed, upon infection, TB mostly targets the host’s macrophages, within which they establish a replicative niche. In reaction, the immune system targets TB in the macrophages’ phagolysosomes, leading to the pathogen’s elimination. In its active state, the *LRRK2* gene has been reported to have a negative impact on the TB outcome by delaying the maturation of phagosomes in macrophages, acting potentially by the recruitment of Class III phosphatidylinositol-3 kinase complex and Rubicon^38,39^. Accordingly, LRRK2 deficiency led to a clear decrease in the TB impact on mice. Compellingly, the missense variant observed in this gene was absent in the NRes group but found at an allelic frequency of 0.6 in the Res group. The *LRRK2* missense variant in the Res group could thus contribute to the bTB resistance phenotype and functional validation of this variant in further study might be decisive in deciphering bTB resistance.

The other candidate genes also have well-documented effects on the immune system. The SHC adaptor protein 3 (*SHC3*) gene is part of the SHC (Src Homology and Collagen) family, which transmits the extracellular signal recognized by the CD16 receptor of the NK cells. In turns, this signal activates different intracellular signaling pathways, among which the MAPK pathway, to release cytokines related to NK cell-mediated cytotoxicity^40–42^. The missense variant, exclusively observed in NRes breeds with an allelic frequency of 0.6, warrants further investigation regarding its impact on managing *Mycobacterium bovis* infection, particularly in modulating inflammation through the *SHC3* gene. Functional validation is crucial to determine its effect, yet the significant finding of this deleterious variant solely in NRes breeds positions it as a compelling candidate for further study.

The remaining deleterious missense variants observed in the *EP300*, *IFNGR1*, *TLR2*, *TLR6*, *IL1A* and *IRAK4* genes all presented a higher allelic frequency in the Res breeds. The P300 proteins, coded by the *EP300* gene, interact with the STAT proteins in the JAK-STAT signaling pathways to control the antigen presentation by the Major Histocompatibility Complexes (MHC) 1 and 2^43–46^. *IFNGR1* which is also a receptor for the JAK-STAT signaling pathway and is one of most frequently reported genes to show an association with TB and bTB susceptibility in human, buffalo, and cattle^47–49^. Toll-like members, which are expressed on the membrane of sentinel cells (such as macrophages, dendritic cells and mast cells) can recognize a wide repertoire of microbes including the 19-kDa lipoprotein and lipoarabinomannan (LAM) of TB^31,50–52^. Especially, *TLR1, TLR2, TLR4*, and *TLR6* in monocytes recruit adaptor proteins such as TIRAP, interleukin-1 receptor associated kinase (IRAK) or the innate immune signal transduction adaptor MyD88 and further activates other components, resulting in the over-expression of pro inflammatory cytokines such as IL-1 and IL-18^53^. TLR2 also contributes to the activation of mast cells after the TB infection^54,55^. Interestingly, polymorphism in *TLR2, TLR4,* and *TLR6* genes has repeatedly been reported to have an association with susceptibility to *Mycobacterium* in cattle and humans^56–61^. Inflammatory cytokine Interleukin 1-alpha, which is secreted after the recognition of *Mycobacterium* by TLRs in monocytes, plays a role in T cell proliferation, chemokine secretion and granuloma formation^62–64^. Polymorphisms of *IL1A* in mice, human, and African buffalo were also modulated susceptibility to tuberculosis^65–68^. The *IRAK4* gene codes for an essential enzyme involved in the TLR pathways. It activates NF-kB and sequentially regulates cytokines and T cell activation^69,70^. In case the *IRAK4* gene is disrupted, the signaling cascade can be interrupted and consequently alter the response to TB^71,72^^.^*.*

The higher prevalence of the deleterious missense variants in these genes observed in the Res breeds might seem counter-intuitive given their role in mediating immunity upon infection. However, as stated above, resistance to tuberculosis depends on a complex balance between pro and anti-inflammatory factors and a better understanding of how the observed variants interact with each other and might contribute to bTB resistance could only be verified through functional validation.

Last, though the* CD48* gene did not display any obvious differences in missense variants, it was both detected through the XP-EHH selection signature and had one of the highest average copy number differences between populations. CD48, combined with the immunoreceptors CD2 and CD244, plays various roles in the activation of cytotoxicity and proliferation of immune cells such as natural killer (NK) cells, B lymphocytes, T lymphocytes, dendritic cells, neutrophils, eosinophils, and mast cells (MC). In particular, the CD48 receptor has a crucial role in the function of NK cells^73–75^, cell type that is reported to directly kill the *Mycobacteria* and activate other immune cells (monocytes, T cells) after TB infection^76–78^. In addition, CD48 has been reported to activate mast cells, leading to the release of proinflammatory mediators controlling the uptake of TB^79^.

## Conclusions

In this study, we propose *LRRK2*, *SHC3, IFNGR1, TLR2, TLR6, IL1A, CD48* and other genes related to the innate immune system as candidate genes for the difference in bTB susceptibility reported between African zebu and European taurine cattle breeds. The *LRRK2* gene appears as a strong candidate as its deficiency has been shown to improve bTB resistance. The results, issued from the screening of WGS data at the base pair resolution, contribute a new layer of information regarding the genetic background of resistance towards bovine tuberculosis. Additionally, these results present potential candidates that warrant further investigation through functional validation. However, this study is limited due to lack of actual infection studies comparing more breeds and individuals. As, the genes reported in this study are expected to work in a connected way in modulating immunity through regulation of the pro- and anti-inflammatory factors, the candidate variants should be integrated in any further validation. After validation, these variants might serve as valuable tools for managing bTB infections in herds through future selective breeding programs.

## Methods

### Data description

The sequences were obtained from the publicly available database of NCBI under the following accession numbers: PRJNA574857 (Arsi, ETB, African buffalo), PRJNA312138 (Kenyan), PRJNA210521 (Holstein), PRJNA318089 (Jersey). Five cattle breeds reported in previous literature for different degrees of resistance to bovine tuberculosis infection and two African buffalo (included as an outgroup in compliance with the recommendations of the population differentiation method FLK) were used in this study. More precisely, the breeds selected as relatively resistant to bTB infection were three African indigenous Zebu breeds (*Bos indicus*) Arsi, Ethiopian Boran, and Kenyan Boran which will be referred to as the Res group throughout the manuscript. The breeds selected as relatively susceptible to bTB were two European *Bos taurus* dairy breeds Holstein, and Jersey. These susceptible breeds will be termed as NRes. A total of ten individuals per breed except for African buffalo (AFB) were used in the comparison. The 52 samples used in this study were part of a previously published larger dataset^80^.

### SNP variant calling

The raw sequence data was processed as described below. First, per-base sequence quality was checked using the fastQC software v.0.11.9^81^. Low quality bases and artifact sequences were removed using Trimommatic v.039^82^. BWA-MEM v.0.7.17^83^ was then used to map high-quality sequence reads to the reference bovine genome (ARS-UCD1.2) with default parameters. Index files for reference and bam files were generated using Samtools v.1.9^84^ and potential PCR duplicates were marked by 'MarkDuplicates' of Picard v.2.20.2^85^. To perform base quality score recalibration (BQSR), 'BaseRecalibrator and 'PrintReads' arguments of genome analysis toolkit (GATK) v.4.40^86^ were used. For masking known sites, variants file (ARS1.2PlusY_BQSR_v3.vcf.gz) from the 1000 Bull genomes Project was used, except for the two AFB samples. The base quality improvement was then checked with the 'AnalyzeCovariates' argument of GATK. Variant calling for AFB was performed as described in the previous paper^80^. Alignment quality and overall alignment statistics were calculated with Sambamba^87^.

To call SNPs from bam files, we used 'HaplotypeCaller' of GATK to create GVCF files. The GVCF files were then combined with 'CombinedGVCFs' of GATK. From combined GVCF files, SNPs were called with 'GenotypeGVCFs' and selected with 'SelectVariants' of GATK, respectively. The 'VariantFiltration' argument was then implemented to avoid possible false-positive calls with following options: cluster size 3; cluster window size 10; SNPs with mean depth (for all individuals) < 1/3 and > 3x (x, overall mean sequencing depth across all SNP sites); quality by depth < 2; phred scaled variant quality score < 30; strand odds ratio > 3; Fisher strand > 60; mapping quality < 40; mapping quality rank sum test < − 12.5; read position rank sum test < − 8. We also removed SNPs with missing genotype rates > 0.01 and non biallelic SNPs. Then with BEAGLE 4.0^88^, genotype refinement, imputation, and phasing were successively applied to the remaining SNPs. Finally, after removing minor allele frequency < 0.01, high-quality SNPs were obtained and annotated with SnpEff v.4.3^89^ for the downstream analysis.

### Analysis of population structure

A set of analyses (3 SNP-based methods and one CNV-based method) were performed to comprehend the population structure of the dataset. First, a Principal Component Analysis (PCA) was performed using the Genome-wide Complex Traits Analysis (GCTA) v.1.93.2^90^. To further investigate the genetic distance between the 5 breeds included in this study, the fixation index FST was computed pairwise between each of the 5 breeds using VCFtools v.4.0^91^, considering 10 kb windows without overlapping. Next, Admixture^24^ was used to determine the most probable number of ancestral populations and assign an ancestral population to each of the 50 individuals. Finally, we drew a CNV based dendrogram among individuals by coding the events in the CNVR regions as -1, 0, and 1 for copy number deletion, no variation, and amplification, respectively. The optimal cluster (K) parameter according to the CNV was found using Gap statistics with nstart 25, K.max 10, B 50 options.

### Detection of selection signatures in SNP

The appearance of bTB having most probably occurred less than 2,000 generations ago, selective sweeps triggered by this pathogen would necessarily be recent or still ongoing. The cross-population extended haplotype homozygosity statistics (XP-EHH), which is based on long haplotype detection, detects recent and ongoing selection having occurred within one of the populations^92^. This method is optimally used for selections having occurred within the last 30,000 years in humans^93^, or 7200 years in equivalent for cattle considering standard approximates of 25- and 6- year generation intervals for the human and bovine species, respectively. XP-EHH was first computed using hapbin 1.3.0^94^ with 10 kb nonoverlapping windows. The average score (absolute value) of all SNPs within a window was used as a summary statistic for each window. To account for SNPs number variation in each window, genomic windows were binned in increments of 200 SNPs with dropping windows containing less than 10 SNPs and combining windows over 600 SNPs into one bin. The empirical *P* value was defined as described in Pickrell et al.^95^: within each bin, for each window *i*, *P* value is the fraction of a window with a value of the statistic greater than that in *i*. The results above *P* value of 0.01 in each bin were taken as candidates.

As a complement, three other methods that might detect selection in time frames occurring before and after the presumed apparition of the bTB causative agent were employed to test for putative ancient selection related to various pathogens exposure and conferring by chance a selective advantage to one of the groups in case of bTB exposure (pre-adaptation).

The cross-population composite likelihood ratio (XP-CLR) method^96^ is one of the statistics developed to detect allele frequency differentiation signature which has been estimated to persist over the equivalent of 18,000 to 12,000 years in cattle. The XP-CLR method was implemented using scripts available at https://github.com/hardingnj/xpclr^97^ , using the following options: –size 10,000; -maxsnps 1000; -minsnps 10. The results were averaged, binned and the *P*-value was defined in the same way informed for XP-EHH.

To make the most insights out of the available data, the commonly used statistic FST, based on population differentiation, was applied with VCFtools v4.0 using the -weir-FST-pop option and setting the -FST-window-size to 10,000. The results were averaged, binned and the P-value was defined according to the afore-mentioned protocol.

Finally, the extended Lewontin and Krakauer (FLK) method^98^ which accounts for the hierarchical structure by using kinship matrix was employed. Two African buffalo were used as outgroups with the default options. The results were averaged, binned and the P-value was defined according to the afore-mentioned protocol.

All the regions selected from methods above were annotated based on the reference genome ARS-UCD1.2 from NCBI RefSeq database^99^. Genes that overlap partly or entirely with the significant regions were defined as candidate genes.

### CNV calling and CNVR identification

CNVs were called with the aligned read depth-based tool CNVnator v0.4.1^100^, using a bin size of 200 bp and filtered with length > 1 kb. The *p*-value was based on t-test statistics < 0.001 and a fraction of reads with zero mapping quality q0 < 0.5 as frequently described in the literature^101,102^. After removing unmatched scaffolds, the 'CNV_overlap.py' script from (https://github.com/bjtrost/TCAG-WGS-CNV-workflow)^103^ was applied to identify copy number variation region (CNVR) as the 50% overlap between remaining CNVs. To minimize false-positives, only CNVRs that were found in more than 3 individuals were kept for downstream analyses^104^.

### Detection of selection signatures in CNV

Two statistical methods were applied for detecting the population difference of CNVR between Res and NRes. First, Vst, a population differentiation estimator similar to FST, was calculated between the two groups as Vst = (Vt − Vs)/Vt with Vt being the total variance of copy number among every individuals, Vs being the average variance within each population weighted by the number of individuals in the population^105^. As small within-variance might generate false-positive CNVs when employing the Vst method, a two sample t-test between the populations was conducted to compare the copy numbers between the two groups (*P*-values adjusted with a Bonferroni correction) as a complement. Regions that were within the top 1% of the highest Vst and which had an adjusted t-test *P*-value below 0.01 were considered as population differentiated CNVRs. Genes overlapping with the CNVR were annotated.

### Selection and characterization of candidate genes

To get more insights into the bovine tuberculosis susceptibility discrepancy between Res and NRes, a rough screening of the annotated genes from the putatively selected regions was performed using two different approaches. First, candidate genes issued from each method which are possibly related to immunity for tuberculosis susceptibility were filtered by implementing gene ontology using the Database for Annotation, Visualization and Integrated Discovery (DAVID)^106^. Overrepresented Gene Ontology (GO) terms and Kyoto Encyclopedia of Genes and Genomes (KEGG) pathways^107^ were searched, using a *P*-value of 0.05 as the threshold for statistical significance. Genes that could be related to tuberculosis susceptibility were kept in further steps.

A literature review was performed as a second approach. The studies based on differentially expressed genes were considered for further steps (Table S1). As a result, a total of 239 genes from the literature that were reported for possible modulation of bTB sensitivity were individually considered as candidate genes and proceeded to the last selection step along with the genes found from the selection signature and GO analysis.

The candidate genes resulting from the SNP-based selection signature filtered with DAVID analysis and the literature-based candidate genes were further refined by keeping the genes whose visual inspection revealed segregation (more than 0.5 difference in either allele frequency) in missense variants between the 2 groups (performed using ARS-UCD1.2). The Sorting Intolerant from Tolerant (SIFT) score^108^ provided by the Ensembl Variant Effect Predictor (VEP) and the Protein Variation Effect Analyzer (PROVEAN) score^109^ were used to determine the outcome of the missense variants on the proteins. The SIFT score is calculated with a normalized probability of observing a different amino acid at a considered position and ranges from 0 to 1 with values comprised between 0 and 0.05 predicted to affect protein function. PROVEAN uses an alignment-based score approach which measures the similarity of a functional sequence and the query sequence. A score threshold of -1.3 was used as it was sufficient to detect the functional changes in the variants of non-human dataset with a balanced accuracy of 75.51%^110^. The strongest candidate genes were visualized with Haploview^111^ to assess the degree of linkage among the selected regions and the adequacy of the window size used in the selection detecting methods (Figs. S2, S3, S4, S5). Haplotype blocks were detected based on the method by Gabriel et al.^112^.

The CNV-related genes filtered with the DAVID analysis were also examined together with the literature genes and candidate genes that showed more than one average copy number difference (Table S4) were further inspected by visualizing the average copy number in 200 bp windows sliding along the gene.

### Supplementary Information

Supplementary Figure 1.

Supplementary Figure 2.

Supplementary Figure 3.

Supplementary Figure 4.

Supplementary Figure 5.

Supplementary Table 1.

Supplementary Table 2.

Supplementary Table 3.

Supplementary Table 4.

Supplementary Legends.

## Data Availability

The dataset supporting the conclusions of this article is available in the Sequence Read Archive (SRA) repository with the Bioproject accession number PRJNA574857 (https://www.ncbi.nlm.nih.gov/bioproject/PRJNA574857/), PRJNA312138 (https://www.ncbi.nlm.nih.gov/bioproject/prjna312138), PRJNA210521 (https://www.ncbi.nlm.nih.gov/bioproject/PRJNA210521), and PRJNA318089 (https://www.ncbi.nlm.nih.gov/bioproject/PRJNA318089).
