# Supplementary figures and images for: Exploring the genetic factors behind the discrepancy in resistance to bovine tuberculosis between African zebu cattle and European taurine cattle
Source: Sci Rep. 2024 Jan 29;14:2370. doi: 10.1038/s41598-024-52606-2 (PMC10824790; doi:10.1038/s41598-024-52606-2)

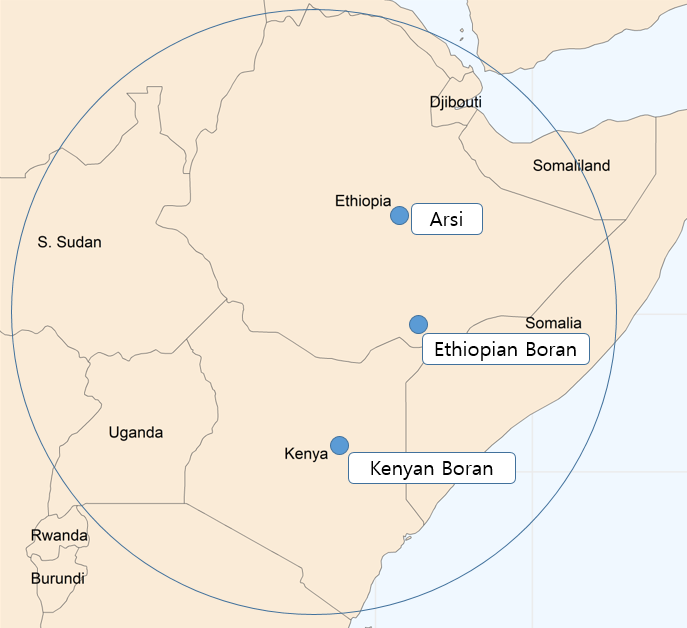

Supplement: Supplementary file 1 — Supplementary Figure 1. [file 41598_2024_52606_MOESM1_ESM.png]

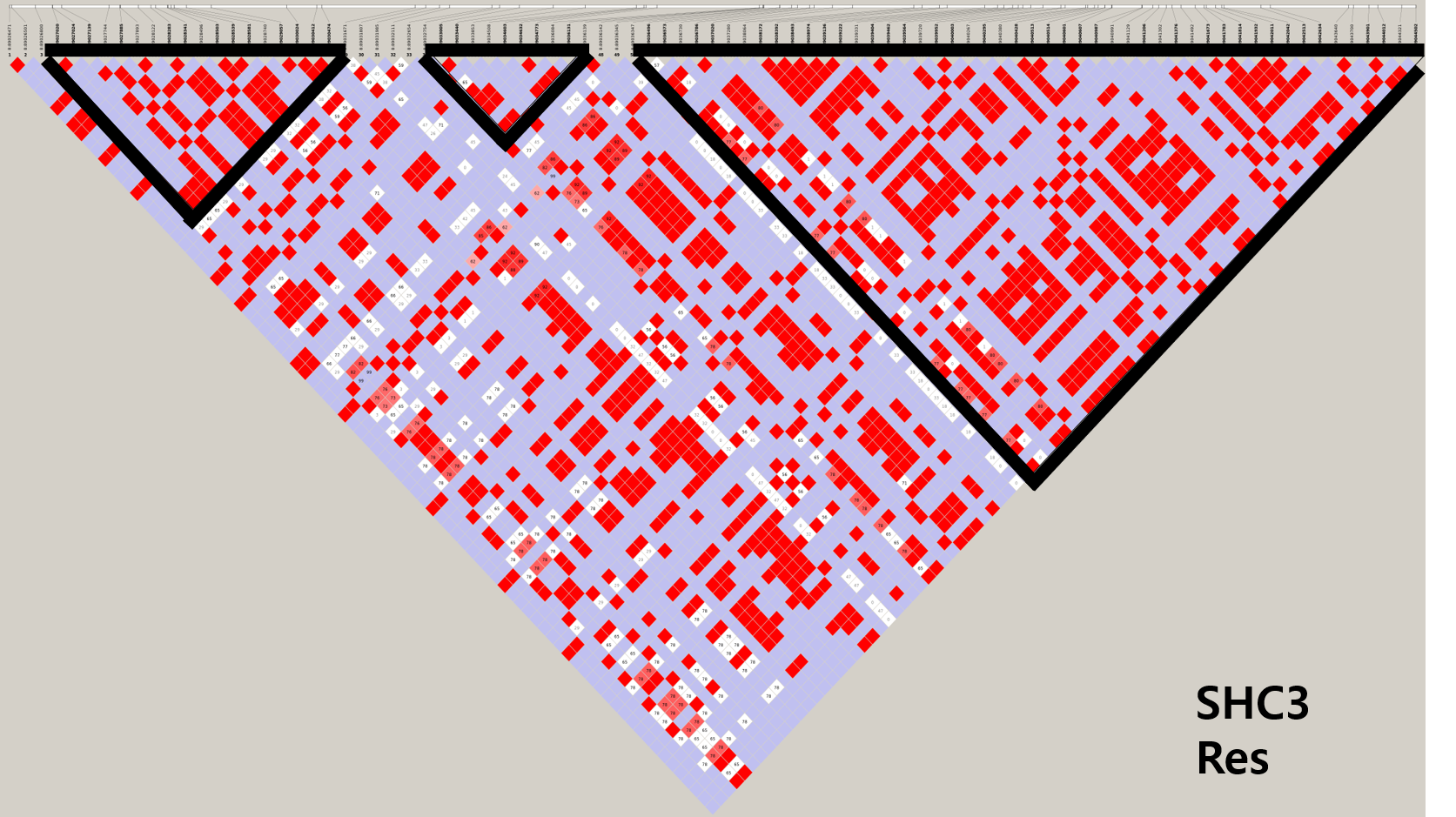

Supplement: Supplementary file 2 — Supplementary Figure 2. [file 41598_2024_52606_MOESM2_ESM.png]

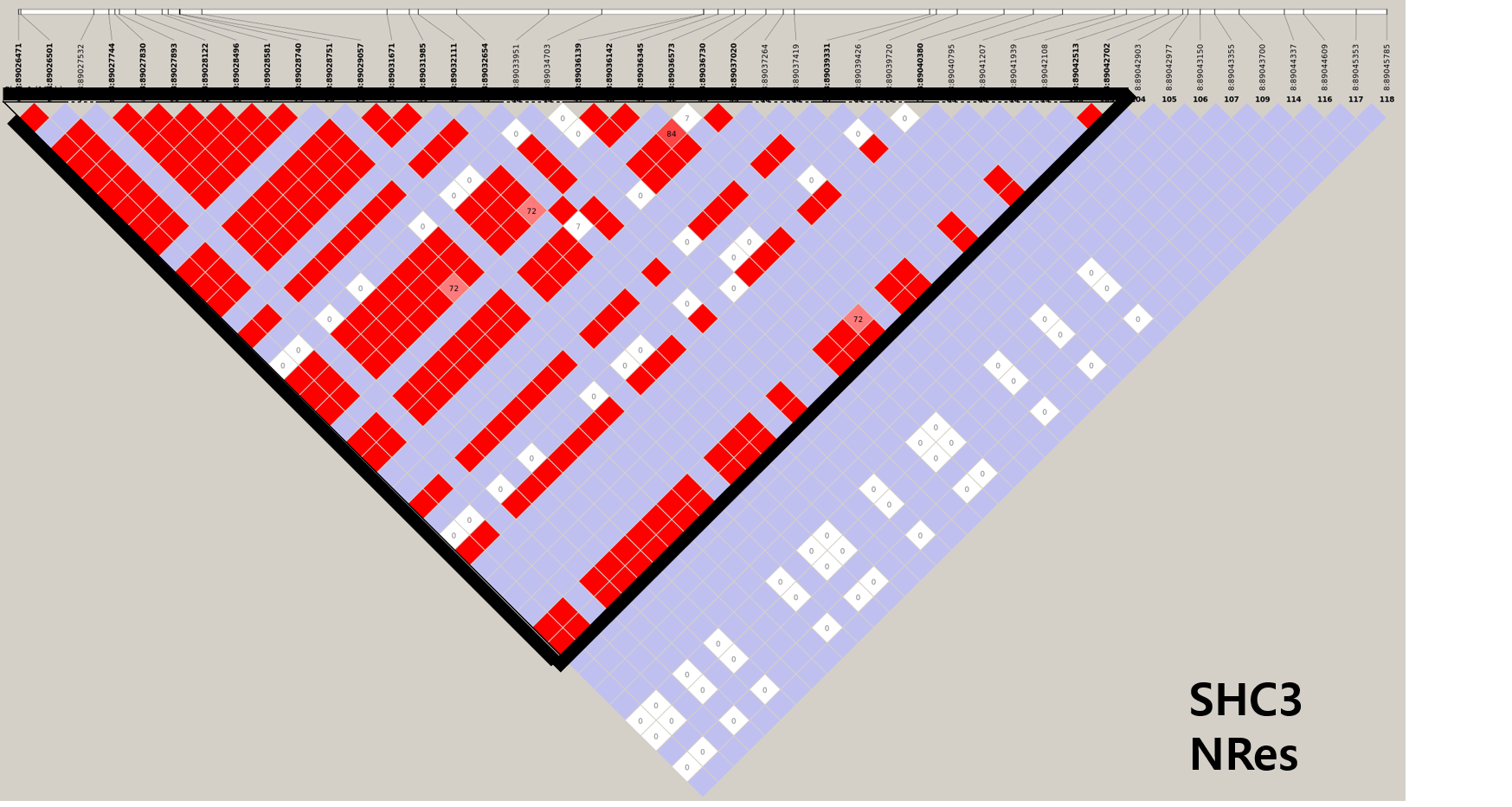

Supplement: Supplementary file 3 — Supplementary Figure 3. [file 41598_2024_52606_MOESM3_ESM.png]

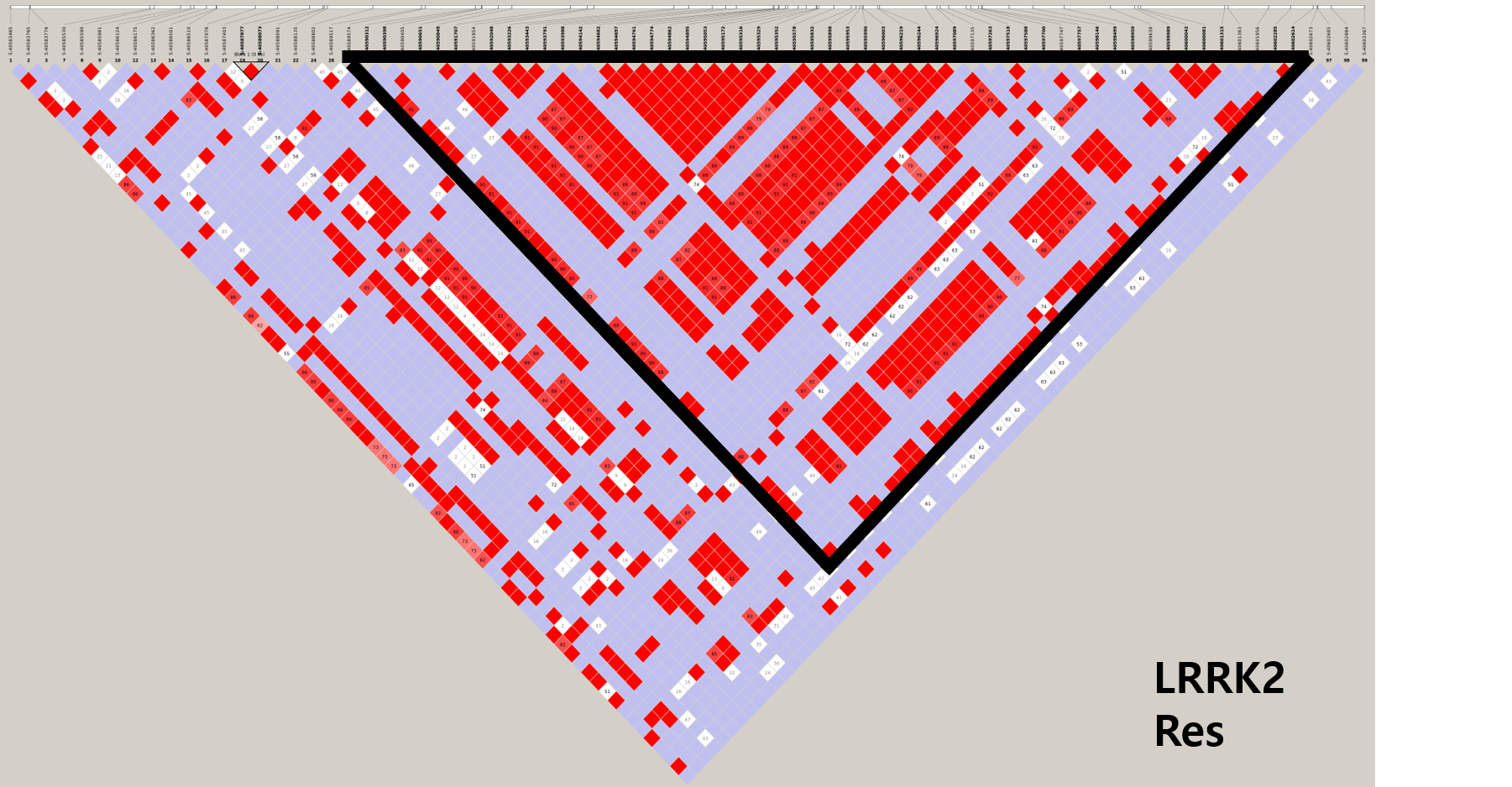

Supplement: Supplementary file 4 — Supplementary Figure 4. [file 41598_2024_52606_MOESM4_ESM.png]

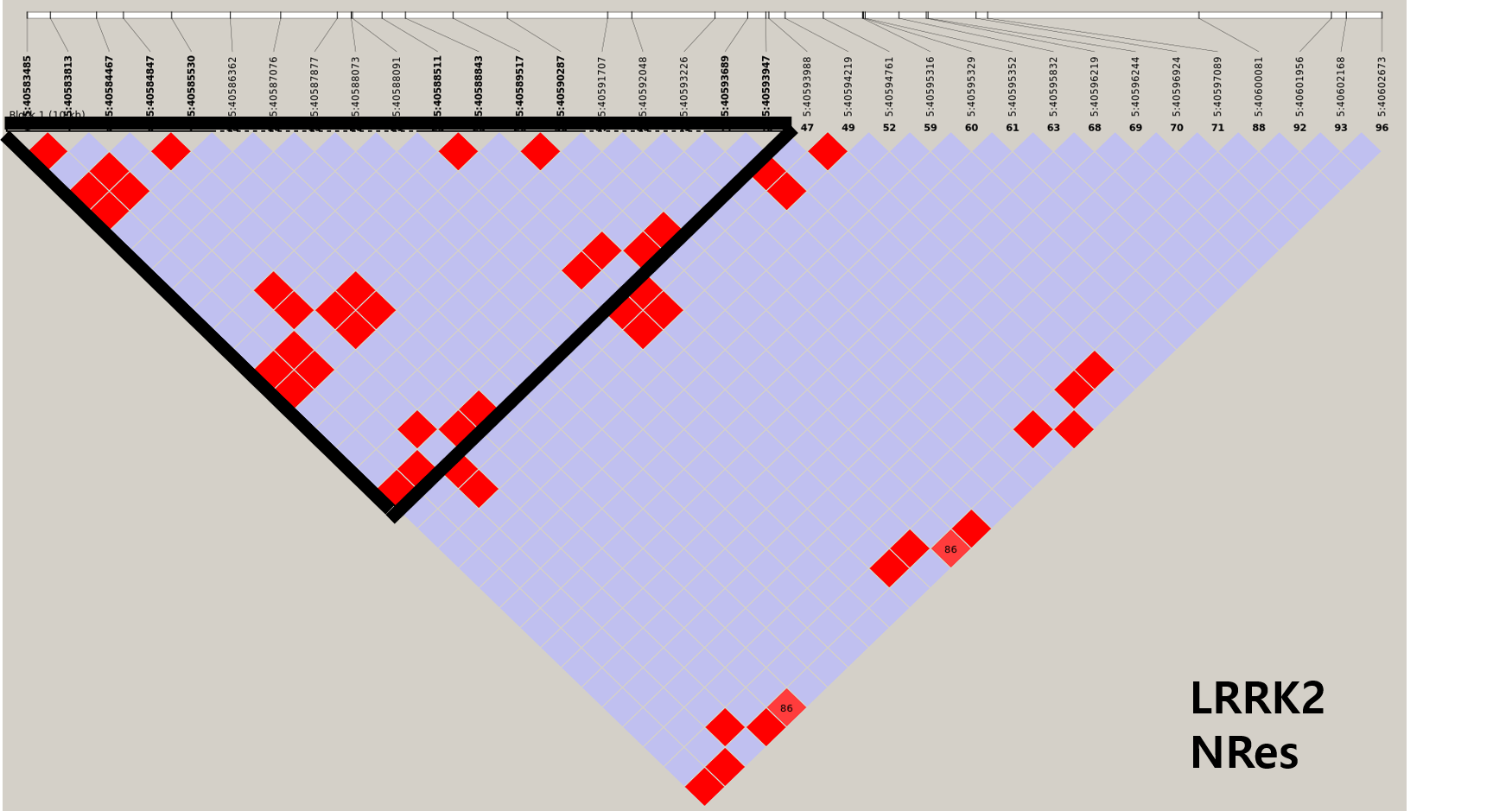

Supplement: Supplementary file 5 — Supplementary Figure 5. [file 41598_2024_52606_MOESM5_ESM.png]
