# Supplementary material for: Exploring the genetic factors behind the discrepancy in resistance to bovine tuberculosis between African zebu cattle and European taurine cattle
Source: Sci Rep. 2024 Jan 29;14:2370. doi: 10.1038/s41598-024-52606-2 (PMC10824790; doi:10.1038/s41598-024-52606-2)
Supplement: Supplementary file 6 — Supplementary Table 1. [file 41598_2024_52606_MOESM6_ESM.docx]

| **Publication** | **Genes** |
| --- | --- |
| Meade et al., 2006 | *BLR1(CXCR5), BOLA-DMA, CD84, IFNG, IL2RB, ITGAV, MGAT2, MMP9, NUDT3, SMAP1, TNFSF13B* |
| Meade et al., 2007 | *ADAM17, AKT1, BCL2, CCL1, CD81, CD84, CXCR3, EEF1G, FGFR1, GAN, GPR98(ADGRV1), IL16, IL8, MCL1, NCOR1, NFATC4, NFKB1, NFKB1, NRM, NRP1, PHB2, PPP2R5B, PRKCB1(PRKCB), RPS6KB2, SFPQ, STK17B, TBK1, TBK1, TLR2, TLR4, TNF, UCP2, UNC84B(SUN2), ZDHHC19* |
| Blanco et al., 2009 | *IFNG, IL10, IL2, IL4, IL8, TNFA* |
| Driscoll et al., 2011 | *IFNGR1, IL20RA, IL22RA2* |
| Kadarmideen at al., 2011 | *SLC11A1* |
| Killick et al., 2011 | *CASP1, CCL2, CD83, CTLA4, CXCL5, CXCR4, DEFB10, IFNAR1, IFNG, IFNGR2, IL15, IL16, IL18, IL1A, IL8, IRAK4, KIR3DS1, MAPK13, MAPK14, MYD88, PTPN2, STAT1, STAT2, TLR2, TLR3, TLR4, TREM1, TYK2, TYROBP* |
| Aranday-Cortes et al., 2012 | *ADA, CCR6, CXCL10, CXCL9, GALNT3, GZMA, GZMB, IFI47, IFNG, IL17A, IL17RE, IL22, IRF4, KLRK1, LAG3, STAT1* |
| Blanco et al., 2012 | *CD14, IL1R, THBS1, MMP9, FYVE* |
| Finlay et al., 2012 | *SLC6A6* |
| Sun et al., 2012 | *TLR1* |
| Bermingham et al., 2014 | *MYO3B, PTPRT* |
| CHENG et al., 2015 | *ACP2, ACTB, AMOTL1, AP1B1, C1QA, C1QB, C3AR1, CACNG4, CCL3, CCL4, CCNG2, CCR1, CCR3, CCR4, CD14, CD244, CD36, CD38, CD55, CDKN1C, CDKN2D, CFB, CLEC4E, COLEC11, CSF1R, CTSB, CTSL2, DAPP1, DDIT3, EDN1, EHD1, F13A1, FASLG, FN1, FOS, FOSL1, FZD4, GNG4, GZMB, HMPX1.FST, ICOS, IFNG, IGFBP3, IL10, IL12B, IL1RAP, IL2RA, IL7, INHBA, JAM3, KLRK1, LAMP3, LDHA, MAP3K8, MRAS, NUMBL, PDK1, PECAM1, PGD, PLA2G4A, PLK3, PPBP, PTAFR, PTGS2, RAB7B, RASGRP1, SIPA1, SORT1, SPP1, STAT1, TCF7L2, THBD, THBS1, TLR8, TNF, TNFRSF25* |
| Kassahun et al., 2015 | *RHOH, TLR1, TLR6, TLR10* |
| Waters et al., 2015 | *IL17, IL22, LIF, LTA, IL19, IL17A, IL17F, TNF, TBX21, IL27, IRF4, IFNG* |
| Doherty et al., 2016 | *ADRB2, ASB, BHLHE40, CCL4, CD226, CD244, CD69, CD83, CXCR4, DTX1, FASLG, FOS, GADD45B, GATA3, HOPX, HOXA10, IFNG, IL18RAP, IRF4, LAG3, LRRC32, MIF, NFATC1, PAWR, PSMB10, PTGER4, RAG2, RORA, RORC, RUNX3, SOX13, TGIF1, TNFAIP3, TNFRSF18, TNFRSF4, TP53INP1, ZBTB7B* |
| Richardson et al., 2016 | *AP3B1, DEF6, HSF1, KALRN, MAPK13, MAPK14, PTN, RNF185, RUNX1T1, SHARPIN, TAPBP, TRAF4, FKBP5* |
| Raphaka et al., 2017 | *RNF144B* |
| Shukla et al., 2017 | *ADORA3, ADRB3, CCL1, CD40, CD80, CSF2, CXCL6, IFNG, IL12, IL2, IL6, IL8, M-SAA3.2, MAPT, NFKB1, NOS2, SPRY2, TGFBI, TNF, TLR2* |
| Shukla et al., 2018 | *CCL20, CCL3, CCL4, CCR3, CCR5, CSF3, CXCL2, CXCR4, IL1, IL12, IL1B, IL23A, IL6, IL8, MAPT, NFKB1, TLR2, TNF, TRIP10* |
| Palmer et al., 2020 | *CXCL10, CXCL9, IFNG, IL13, IL1A, IL1B, IL21, IL22, TNFA* |
| McLoughlin et al., 2021 | *CDKN1A, CXCR4, EVI2A, FOSB, FRMD6, HBEGF, KRT17, KRT17, NR4A1, NR4A2, OSM, PLAUR, RGS16, SERPINB4, THBD, ZFP36L2, ITK, SERPINB4* |
