# Supplementary material for: Exploring the genetic factors behind the discrepancy in resistance to bovine tuberculosis between African zebu cattle and European taurine cattle
Source: Sci Rep. 2024 Jan 29;14:2370. doi: 10.1038/s41598-024-52606-2 (PMC10824790; doi:10.1038/s41598-024-52606-2)
Supplement: Supplementary file 10 — Supplementary Legends. [file 41598_2024_52606_MOESM10_ESM.docx]

**Behind the discrepancy in susceptibility to bovine tuberculosis: insights from African zebu cattle**

SangJung Lee^1^_,_ Charton Clémentine^1^, Heebal Kim^1^*

^1^Department of Agricultural Biotechnology and Research Institute of Agriculture and Life Sciences, Seoul National University, Seoul 08826, Republic of Korea

* Corresponding author: Heebal Kim

Department of Agricultural Biotechnology and Research Institute of Agriculture and Life Sciences, Seoul National University, Seoul 08826, Republic of Korea

Email Address: heebal@snu.ac.kr

**Supplementary Fig. S1** The geographical origin of the three relatively resistant breeds Arsi, Ethiopian Boran, and Kenyan Boran and putative origin of bovine tuberculosis (region in circle) that were reported from previous studies^1,2^. The figure was drwan with ggplot 3.4.2 (https://ggplot2.tidyverse. org/ ).

**Supplementary Fig. S2** Linkage disequilibrium and haplotype block within 20kb region of SHC3 gene (8:89026345-8:89046345) in Res group viewed by Haploview. Missense variants found from the study is in the center (8:89036345). Thick lines in black indicate the haplotype blocks with sizes of 3kb, 3kb, 8kb from left to right. LD nodes were colored according to *D’* values and the standard Haploview color scheme.

**Supplementary Fig. S3** Linkage disequilibrium and haplotype block within 20kb region of SHC3 gene (8:89026345-8:89046345) in NRes group viewed by Haploview. Missense variants found from the study is in the center (8:89036345). Thick lines in black indicate the haplotype blocks with size of 16kb. LD nodes were colored according to *D’* values and the standard Haploview color scheme.

**Supplementary Fig. S4** Linkage disequilibrium and haplotype block within 20kb region of LRRK2 gene (5:40583443-5:40603443) in Res group viewed by Haploview. Missense variants found from the study is in the center (5:40593443). Thick lines in black indicate the haplotype blocks with size of 12kb. LD nodes were colored according to *D’* values and the standard Haploview color scheme.

**Supplementary Fig. S5** Linkage disequilibrium and haplotype block within 20kb region of LRRK2 gene (5:40583443-5:40603443) in Res group viewed by Haploview. Missense variants found from the study is in the center (5:40593443). Thick lines in black indicate the haplotype blocks with size of 10kb. LD nodes were colored according to *D’* values and the standard Haploview color scheme.

**Supplementary Table S1.** Summary of the genes identified as related to bovine tuberculosis through literature review

Supplementary Tables 2, 3 and 4 are in individual files.

**Supplementary Table S2.** Sample information and alignment statistics

**Supplementary Table S3.** David Go and Kegg pathway terms on selection signature results**.**

**Supplementary Table S4.** Average Copy number differences and overlapping gene information of CNVR regions.

**Reference**

1. Loiseau, C. *et al.* An African origin for Mycobacterium bovis. *Evol Med Public Health* **2020**, 49 (2020).

2. Kim, K. *et al.* The mosaic genome of indigenous African cattle as a unique genetic resource for African pastoralism. *Nature Genetics 2020 52:10* **52**, 1099–1110 (2020).
